# Construction and Evaluation of the Tumor-Targeting, Cell-Penetrating Multifunctional Molecular Probe iCREKA

**DOI:** 10.1155/2018/7929617

**Published:** 2018-03-04

**Authors:** Li-juan Wang, Hong-sheng Li, Quan-shi Wang, Hu-bing Wu, Yan-jiang Han, Wen-lan Zhou, Meng Wang, Shun Huang

**Affiliations:** PET Center, Nanfang Hospital, Southern Medical University, Guangzhou, Guangdong 510515, China

## Abstract

A novel tumor stroma targeting and membrane-penetrating cyclic peptide, named iCREKA, was designed and labeled by fluorescein isothiocyanate (FITC) and positron emitter ^18^F to build the tumor-targeting tracers. The FITC-iCREKA was proved to have significantly higher cellular uptake in the glioma U87 cells in the presence of activated MMP-2 than that in absence of activated MMP-2 by cells fluorescence test* in vitro*. The tumor tissue fluorescence microscope imaging demonstrated that FITC-iCREKA accumulated in the walls of the blood vessels and the surrounding stroma in the glioma tumor at 1 h after intravenous injection. While at 3 h after injection, FITC-iCREKA was found to be uptaken in the tumor cells. However, the control FITC-CREKA can only be found in the tumor stroma, not in the tumor cells, no matter at 1 h or 3 h after injection. The whole-animal fluorescence imaging showed that the glioma tumor could be visualized clearly with high fluorescence signal. The microPET/CT imaging further demonstrated that 18F-iCREKA could target U87MG tumor* in vivo* from 30 min to 2 h after injection. The present study indicated the iCREKA had the capacity of tumor stroma targeting and the membrane-penetrating. It was potential to be developed as the fluorescent and PET tracers for tumor imaging.

## 1. Introduction

Currently, the diagnosis and treatment of malignant tumors remain extremely difficult. Molecular imaging and high-precision targeted therapy represent important directions for the future development of cancer therapy. Molecular recognition is the cornerstone of molecular imaging and targeted therapy. The construction of molecular probes that not only are capable of recognizing specific molecular targets in tumor tissues but also possess the ability to penetrate into tumor cells is of great significance for the specific diagnosis of tumors. Constructing such molecular probes is even more important for specific targeted therapies that need to overcome the biological barrier effect of the cell membrane.

Cell-penetrating peptides (CPPs), also known as protein transduction domains (PTDs), are a special class of polypeptides that have the ability to overcome the physiological barrier imposed by the cell membrane. These peptides can freely penetrate the cell membrane and enter the cytoplasm through endocytosis or specific channels. In addition, CPPs may serve as carriers, carrying exogenous macromolecules up to 100 times their molecular weight (such as drugs or imaging agents) into cells. Therefore, CPPs have been used as important vectors to deliver imaging agents or therapeutic drugs into cells. However, conventional CPPs have no tumor-targeting activity, entering tumor cells and normal cells with equal ease [1–13]. Numerous studies have been carried out to develop CPPs that possess both tumor-targeting and cell-penetrating properties. In particular, conventional CPPs have been chemically modified and screened to identify CPPs capable of binding to specific targets (such as tLyP-1 and RGD) [14–20]. However, in general, broad-spectrum tumor-specific targeting is extremely difficult to achieve.

**Figure 1:**
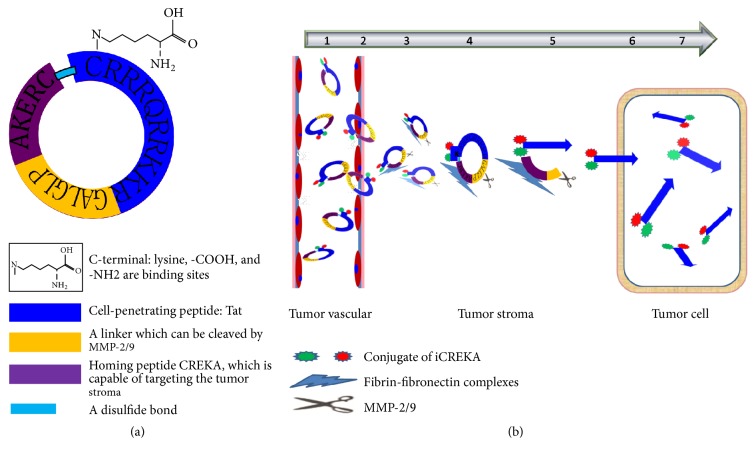
The structure (a) and the mechanism of action (b) of iCREKA.

To this end, a novel multifunctional polypeptide molecular probe that possesses both tumor-targeting and cell-penetrating properties, iCREKA, was designed in the present study. The probe was constructed by connecting the following 3 main parts (Figure 1): the homing peptide CREKA, which is capable of targeting the tumor stroma, the cell-penetrating peptide Tat, and a linker (PLGLAG) that can be cleaved by matrix metalloproteinase-2 and -9 (MMP-2/9). To improve the stability of iCREKA in the blood, the 2 terminal cysteine (Cys) residues in the homing peptide and the membrane-penetrating peptide were covalently linked via a disulfide bond, forming a cyclic peptide. In addition, a lysine residue was added to the C-terminus of the cyclic peptide, which was used for fluorescein or radionuclide labeling.

The cyclic peptide iCREKA reaches tumor tissues via blood circulation. CREKA is expected to bind specifically to the fibrin-fibronectin complexes that are widely and abundantly distributed in tumor stroma but rarely present in normal tissues. Tumor tissues also overexpress MMP-2/9 and hydrolytic enzymes. MMP-2/9 recognize and cleave iCREKA between CREKA and the membrane-penetrating peptide, while hydrolytic enzymes hydrolyze the disulfide bond. As a result, the fluorescent or radionuclide-labeled membrane-penetrating peptide is released, which penetrates the plasma membrane and enters tumor cells. Since normal tissues express no or low levels of fibrin-fibronectin complexes and MMP-2/9, the cyclic peptide probe iCREKA should not be accumulated and be cleaved in normal tissues. Consequently, iCREKA is unable to enter normal cells. Thus, tumor-specific targeting and cell penetration are achieved (Figure 1).

In order to verify whether the cyclic peptide iCREKA specifically targets tumor tissues and possesses cell-penetrating function, the present study used CREKA as a control peptide and employed a fluorescent labeling approach to investigate the differences between iCREKA and CREKA at the cell, tissue, and whole-animal levels. In addition, the present study attempted to label iCREKA using the positron-emitting nuclide fluorine-18 (^18^F) and examine the* in vivo* tumor-targeting activity of labeled iCREKA using live-animal positron emission tomography (PET)/computerized tomography (CT) imaging. The results are reported below.

## 2. Materials and Methods

### 2.1. Materials

^18^F was produced in our center using the PETtrace accelerator (GE Healthcare, USA). iCREKA, fluorescein isothiocyanate-conjugated iCREKA (FITC-iCREKA), and the control peptides CREKA and FITC-CREKA were custom-synthesized at Shanghai Qiangyao Biological Technology Co., Ltd. The anti-fibrinogen antibody was purchased from Abcam, UK (number: ab34269). The anti-MMP-2 antibody was purchased from ABclonal, America (number: A1558). Cyanine 3- (Cy3-) labeled goat anti-rabbit IgG and 4′,6-diamidino-2-phenylindole dihydrochloride (DAPI) were purchased from Beyotime Biotechnology Co., Ltd.

### 2.2. Tumor Cell Lines

A human glioma cell line U87 was used in this study, and it was purchased from the Institute of Biochemistry and Cell Biology, Shanghai Institutes for Biological Sciences, Chinese Academy of Sciences (Shanghai, China). Cells were cultured at 37°C in a humidified 5% carbon dioxide-containing atmosphere, using Dulbecco's modified Eagle's medium (DMEM) (Hyclone, America) supplemented with 10% fetal calf serum (Hyclone, America).

### 2.3. Animal Model

Animal experiments were conducted under a protocol approved by the Nanfang hospital animal ethics committee at the Southern Medical University (Application number NFYY-2011-126). All surgery was performed under isoflurane anesthesia, and all efforts were made to minimize suffering.

The Laboratory Animal Center at the Southern Medical University provided female BALB/C athymic nude mice (nude mice) 4–6 wk of age. Glioma xenografts (U87 cells) were transplanted into subcutaneous of nude mice by injecting 1 × 10^6^ cells intramuscularly into the right flank. Tumor xenografts were monitored until the largest tumor diameter was approximately 0.5–1 cm, which took 2-3 weeks. Forty mice were inoculated, and models of human glioma in nude mice were made successful in 32 mice. The success rate was 80%.

### 2.4. Chemical Synthesis of the Cyclic Peptide iCREKA and the Fluorescent Probes FITC-iCREKA and FITC-CREKA

The linear polypeptide CREKAPLGLAGRKKRRQRRRC was synthesized using solid-phase peptide synthesis (SPPS) [21]. Subsequently, cyclization was performed between the two Cys residues located at the N- and C-termini of the peptide. A lysine residue was then added to the C-terminus of the cyclized peptide, generating the cyclic peptide iCREKA. To prepare the fluorescent probe FITC-iCREKA, FITC was coupled to the amine group of the lysine side chain.

The control peptides CREKA and FITC-CREKA were synthesized using the same approach.

### 2.5. Cytotoxicity

The cytotoxicity of iCREKA was examined in U87 glioma cells using the Cell Counting Kit-8 (KeyGen Biotech) according to the instruction of the manufacturer. Cells were seeded at a density of 1 × 10^4^ cells/well in 96-well plates, allowed to grow for 24 h, and exposed to various concentrations of iCREKA for 24 h. The absorbance of each well at 450 nm was measured with an absorbance microplate reader (BIOTEK ELX800, USA). The cell viability was calculated by

(1) Viability  %=Absorbancetest−AbsorbanceblankAbsorbancecontrol−Absorbanceblank×100%.

### 2.6. The Membrane Penetration Effect of FITC-iCREKA after Cleavage by MMP-2

(1) Examination of the binding of FITC-iCREKA to U87 glioma cells after MMP-2 addition.

(a) MMP-2 activation: to obtain active MMP-2, 80 *μ*l of 0.7 mg/ml mouse MMP2/Tris-HCl solution was mixed with 0.1 ml of 2.5 mmol/l4-aminophenylmercuric acetate (APMA) and incubated for 2 h in a 37°C water bath.

(b) Fluorescence examination: one hundred microliters of active MMP-2/1640 solution (0.7 mg/ml) was mixed with 0.9 ml of freshly prepared serum-free cell culture medium containing FITC-iCREKA (10 *μ*M). Following filter sterilization, 1 ml of the mixture was added to cell culture dishes containing exponentially growing U87 glioma cells. After incubation at 4°C for 60 min, U87 cells were washed with phosphate-buffered saline (PBS, 5 min × 3 times), fixed with 75% ethanol, and stained with DAPI. Fluorescence distribution in live cells was examined on a confocal microscope.

U87 cells were seeded into 96-well plates at a density of 1 × 104 per well and grew to confluence over 24 h. The cells were incubated with 10 *μ*M of FITC-iCREKA with activated MMP-2 as test group and 10 *μ*M of FITC-iCREKA without activated MMP-2 as control one for 1 h at 37°C. After the incubation, the culture media were removed and the cells were washed twice using the PBS. The cells were then trypsinized, centrifuged (4°C, 5 min, 1000 rpm), and diluted to 1 × 106 cells/mL. The test and control samples were analyzed on a flow cytometer (Becton Dickinson, Oxford, UK) to measure the cellular fluorescence.

(2) Examination of the binding of FITC-iCREKA to U87 glioma cells without MMP-2 addition.

FITC-iCREKA (the same amount as above) was sterilized by filtration. Subsequently, 1 ml of the FITC-iCREKA solution was added to U87 glioma cells. After incubation at 4°C for 60 min, U87 cells were washed 3 times with PBS (5 min each). The fluorescence distribution in live cells was examined on a confocal microscope. The intracellular fluorescence intensity, which reflected the level of FITC-iCREKA uptake, was analyzed using flow cytometry.

(3) Examination of the binding of the control peptide FITC-CREKA to U87 glioma cells.

The binding between U87 cells and the control peptide FITC-CREKA was assayed using the same approach. The fluorescence distribution was examined using confocal microscopy. The intracellular fluorescence intensity, which reflected the level of FITC-CREKA uptake, was analyzed using flow cytometry.

### 2.7. Expression of Fibrin and MMP-2 in Tumor Tissues

Tumor samples from U87 tumor xenografts were examined for fibrin and MMP-2 expression using an immunofluorescent and an immunohistochemical staining. Tumor sliders were firstly handled using rabbit anti-fibrinogen antibody or anti-MMP-2 antibody as the primary antibody. Then Cy3-labeled or horseradish peroxidase enzyme-labeled polymers conjugated to anti-rabbit immunoglobulins as the secondary antibody were added to react with the primary antibody. The dilution factors for primary antibody were 1 : 1000 for antifibrin and 1 : 100 for anti-MMP-2. The images of fibrinogen and MMP-2 expression in tumor tissues were acquired via a fluorescent microscope and immunohistochemical staining.

### 2.8. The Targeting and Cell-Penetrating Activities of FITC-iCREKA in Tumor Tissues

Tumor-bearing mice were randomly divided into the experimental group (*n* = 6) and the control group (*n* = 6). The experimental group and the control group received FITC-iCREKA and FITC-CREKA, respectively (1 mM, 150 *μ*l), via tail-vein injection. Subsequently, tumor tissues were collected from each group at multiple time points. The collected tumor tissues were frozen, sectioned, and stained with DAPI. The distribution of FITC-iCREKA and FITC-CREKA in tumor tissues was observed and compared at each time point using confocal microscopy.

### 2.9. The Tumor-Targeting Activity of FITC-iCREKA in Tumor-Bearing Mice

Tumor-bearing mice were randomly divided into the experimental group (*n* = 6) and the control group (*n* = 6), which received FITC-iCREKA and FITC-CREKA, respectively (1 mM, 150 *μ*l), via tail-vein injection. At 1 h after injection, tumors and various organs were isolated, washed, and imaged using the Kodak* in vivo* imaging system F. Fluorescence distribution was examined in tumors and in various organs and tissues. Images were analyzed using the built-in Kodak Molecular Imaging (MI) software. Regions of interest (ROIs) were outlined on the fluorescence images along the edges of the tumors and various tissues. Fluorescence counts in each ROI were determined, and the tumor/nontumor uptake ratios of FITC-iCREKA and FITC-CREKA were calculated by comparing the corresponding fluorescence counts in tumor and normal tissues.

### 2.10. The Tumor-Targeting Activity of ^18^F-iCREKA in Tumor-Bearing Mice

^18^F-FP-iCREKA was synthesized using the method developed by Chin et al. [22], which involved multiple reactions. The chemical and radiochemical purities of ^18^F-FP-iCREKA were determined by high-performance liquid chromatography (HPLC) and thin layer chromatography (TLC) combined with radioactivity detection.

Tumor-bearing mice received 5.50–7.40 MBq (150–200 *μ*Ci) of the imaging agent ^18^F-iCREKA via tail-vein injection and were anesthetized using isoflurane. The mice were then subjected to microPET/CT imaging at 30, 60, and 120 min after ^18^F-iCREKA injection. The uptake of ^18^F-iCREKA by tumors and various tissues was examined.

ROIs were outlined on the PET images using the Syngo image analysis software installed on the microPET/CT system. The radioactivity counts were determined, and the tumor/brain ratio was calculated.

### 2.11. Statistical Analysis

The results were analyzed using the SPSS 20.0 statistical software. Measurement data were presented as the mean ± standard deviation. The Kolmogorov-Smirnov (K-S) test was employed to examine the normality of the data. The independent two-sample *t*-test was conducted to compare the fluorescent intensity and tumor/nontumor ratios between the experimental group and the control group. *P* values less than 0.05 were considered statistically significant.

## 3. Results

### 3.1. Chemical Synthesis of the Cyclic Peptide iCREKA and the Fluorescent Probes FITC-iCREKA and FITC-CREKA

The peptides iCREKA and CREKA were chemically synthesized using the SPPS approach. The fluorescent probes FITC-iCREKA and FITC-CREKA were prepared using FITC, a derivative of fluorescein. The calculated molecular weights are 2665.26 for iCREKA, 3169.81 for FITC-iCREKA, and 995.09 for FITC-CREKA, respectively. The measured molecular weights of the peptides are [M + H]+. The ESI-HRMS *m*/*z* [M + H] were found to be 2665.6 for iCREKA, 3170.4 for FITC-iCREKA and 994.6 for FITC-CREKA, respectively. HPLC analysis demonstrated that the purity of iCREKA, FITC-iCREKA, and FITC-CREKA was 98.27%, 98.23%, and 98.66%, respectively, after purification.

### 3.2. Cytotoxicity

**Figure 2:**
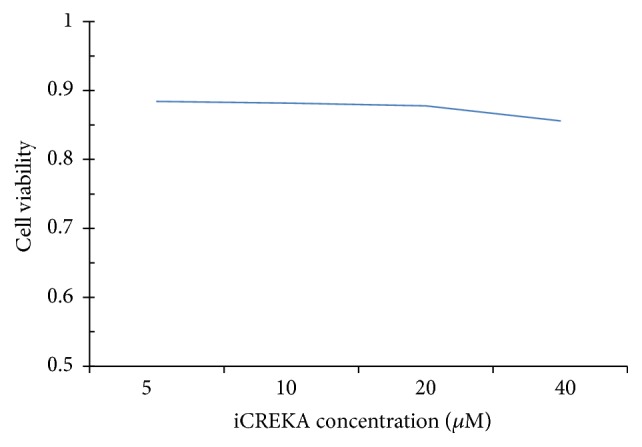
Cytotoxicity of iCREKA at various concentrations.

The cell viability was only slightly damaged by the iCREKA. The cell viability was approximately 88.0% when the concentration of added iCREKA was 10 *μ*M, which was the added concentration of iCREKA in the cell-penetrating experiments (Figure 2).

### 3.3. FITC-iCREKA Penetrates the Cell Membrane and Enters Tumor Cells after Cleavage by MMP-2

**Figure 3:**
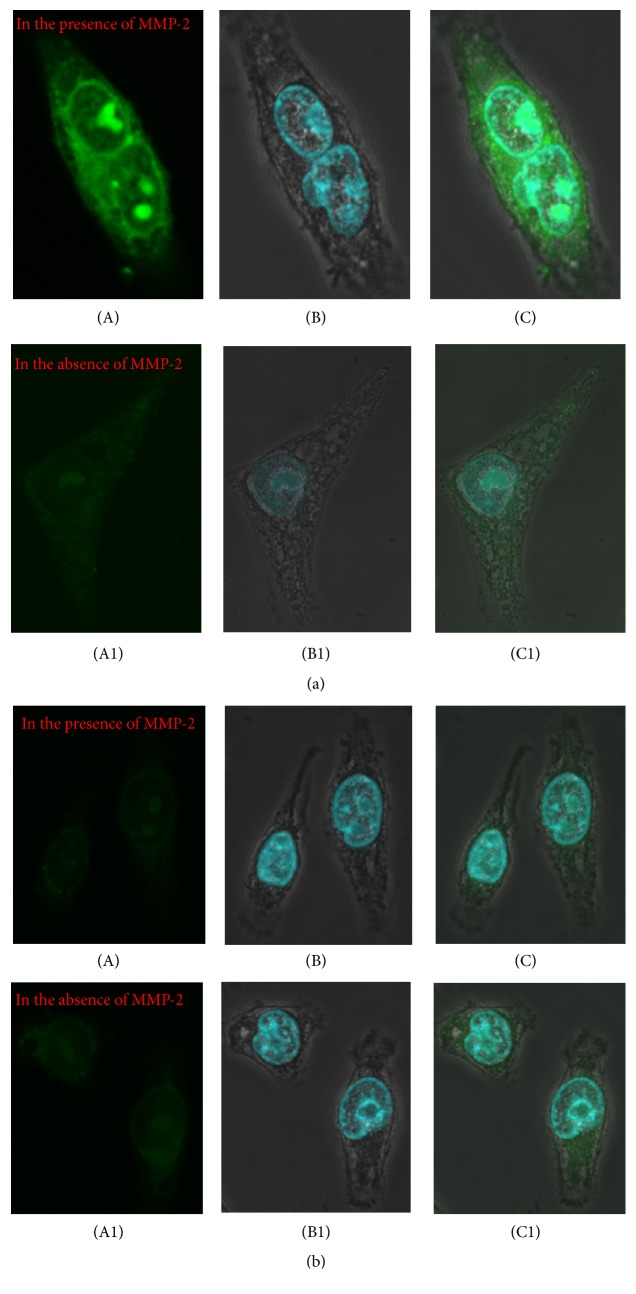
Fluorescent confocal imaging of cell internalization of FITC-iCREKA and FITC-CREKA in the fixed U87 glioma cells ((a) and (b)). (a) Cell internalization of FITC-iCREKA in the presence and in the absence of MMP-2 (green, (A), (A1)), staining of nucleus (blue, (B), (B1)), and merged image of FITC-iCREKA uptake and nucleus ((C), (C1)). (b) Cell internalization of FITC-CREKA in the presence and in the absence of MMP-2 (green, (A), (A1)), staining of nucleus (blue, (B), (B1)), and merged image of FITC-CREKA uptake and nucleus ((C), (C1)). Magnification: ×60.

**Figure 4:**
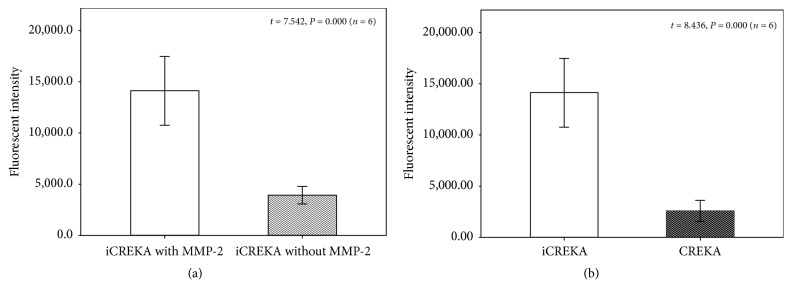
(a) Fluorescent intensity of FITC-iCREKA with and without activated MMP-2 in U87 cells using the flow cytometry analysis. (b) Fluorescent intensity of FITC-iCREKA and FITC-CREKA in U87 cells using the flow cytometry analysis.

The cell-penetrating activity of FITC-iCREKA was examined in the presence and absence of activated MMP-2. In the presence of activated MMP-2, we found that accumulation of green fluorescence within the tumor cells was intense, and the green fluorescence accumulated not only into the cytoplasm, but also into the nucleoli. of U87 cells. However, in the absence of activated MMP-2, the uptake of green fluorescence within the tumor cells was minimal (Figure 3). Flow cytometry indicated that the addition of activated MMP-2 to the culture medium resulted in significantly increased FITC-iCREKA uptake into U87 cells (the fluorescence intensities in the presence and absence of activated MMP-2 were 14110.0 ± 3205.65 and 3921.33 ± 821.27, resp., *t* = 7.542, *P* = 0.000) (Figure 4(a)).

The cell-penetrating activity of the control peptide FITC-CREKA was examined in the presence and absence of activated MMP-2. Only extremely low levels of green fluorescence were detected in the plasma membrane of U87 cells and inside U87 cells, regardless of the presence of activated MMP-2 in the culture medium (Figure 3).

Flow cytometry analysis revealed that FITC-iCREKA uptake was significantly higher than FITC-CREKA uptake in U87 cells (14110.0 ± 3205.65 versus 2569.50 ± 975.84, *t* = 8.436, *P* = 0.000) (Figure 4(b)).

### 3.4. Fibrin and MMP-2 Are Highly Expressed in Tumor Tissues

**Figure 5:**
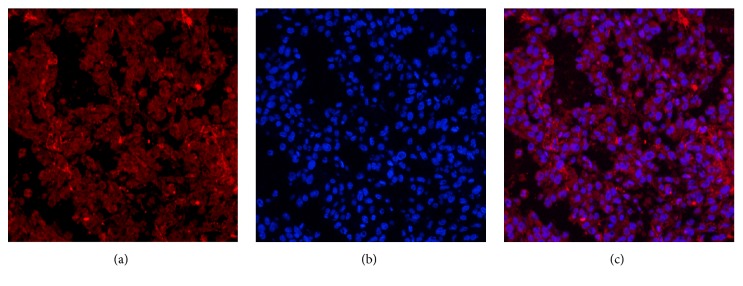
Immunofluorescent staining of the fibrin expression in the tumor tissues. (a) The immunofluorescent staining of the fibrin expression in the tumor tissues (red). (b) DAPI staining of cell nuclei (blue). (c) Fusion of (a) and (b). Positive expression of fibrin was detected in U87 tumor tissues. (magnification: ×200; scale bars, 3 *μ*m).

**Figure 6:**
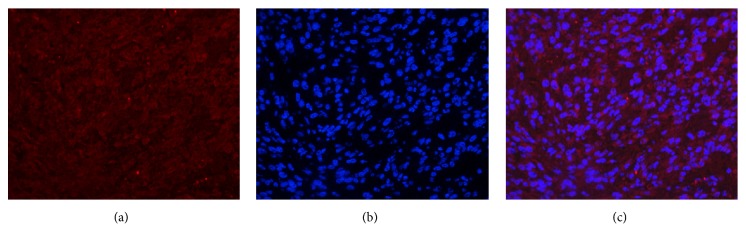
Immunofluorescent staining of the MMP-2 expression in the tumor tissues. (a) The immunofluorescent staining of the MMP-2 expression in the tumor tissues (red). (b) DAPI staining of cell nuclei (blue). (c) Fusion of (a) and (b). Positive expression of MMP-2 was detected in U87 tumor tissues. (magnification: ×200; scale bars, 3 *μ*m).

**Figure 7:**
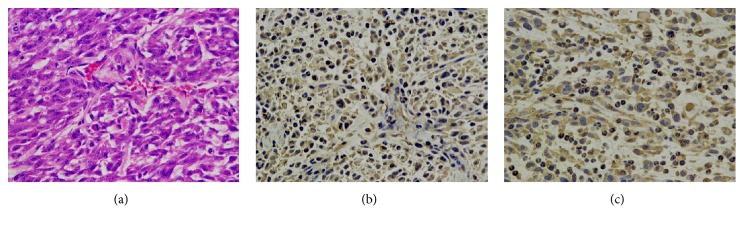
Immunohistochemical staining of the fibrin and MMP-2 expression in the tumor tissues. (a) The HE staining of tumor tissue. (b, c) Immunohistochemical staining of fibrin and MMP-2 expressions in tumor tissue (brown). Positive expressions of fibrin and MMP-2 were detected in U87 tumor tissues. (magnification: ×200; scale bars, 3 *μ*m).

Immunofluorescent and an immunohistochemical staining demonstrated that fibrin and MMP-2 were highly expressed in tumor tissue (Figures 5–7).

### 3.5. The Targeting and Cell-Penetrating Activities of FITC-iCREKA in Tumor Tissues

**Figure 8:**
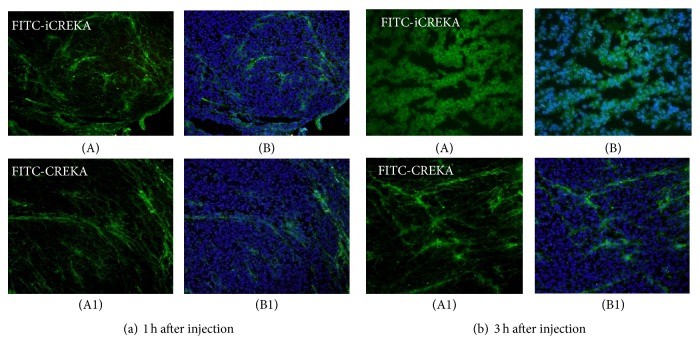
Fluorescence distribution in tumor tissues at 1 h (a) and 3 h (b) after tail-vein injection of FITC-iCREKA (A) and FITC-CREKA (A1) into model mice bearing U87 gliomas. (A) FITC-iCREKA; (A1) FITC-CREKA; (B) and (B1) merged images showing FITC-iCREKA/FITC-CREKA and the nucleus. Magnification: ×200.

Tumor-bearing mice received FITC-iCREKA or the control fluorescent probe FITC-CREKA via tail-vein injection. Fluorescence imaging revealed that FITC-iCREKA accumulated mainly within the stroma of tumor tissues at 1 h after tail-vein injection. In addition, FITC-iCREKA showed the same distribution as that of fibrinogen. At 1 h after tail-vein injection, FITC-CREKA also accumulated in the tumor stroma and exhibited essentially the same distribution as FITC-iCREKA (Figure 8).

Three hours after tail-vein injection of FITC-iCREKA or the control fluorescent probe FITC-CREKA into the tumor-bearing mice, fluorescence imaging analysis revealed that FITC-iCREKA accumulated in tumor cells. In contrast, the control fluorescent probe FITC-CREKA remained in the tumor stroma. No significant uptake of FITC-CREKA fluorescence was detected in tumor cells (Figure 8).

### 3.6. The Tumor-Targeting Activity of FITC-iCREKA in Tumor-Bearing Mice

**Figure 9:**
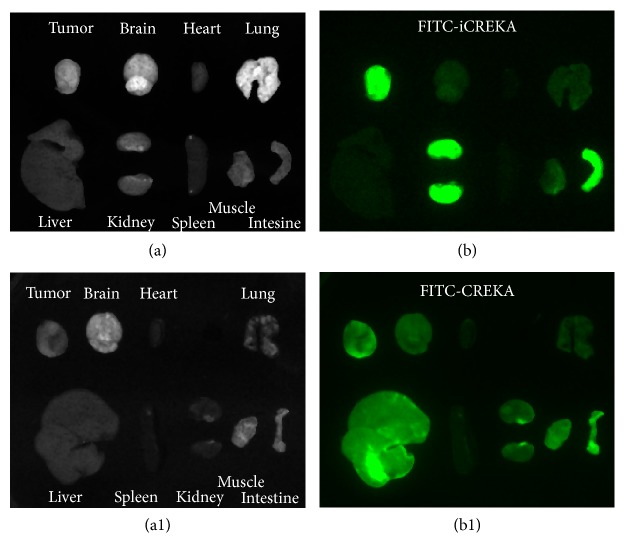
*In vivo* fluorescence imaging of tumors and normal organs at 1 h after tail-vein injection of nude mice bearing U87 gliomas with FITC-iCREKA (a and b) and FITC-CREKA (a1 and b1). Gallbladder was excluded from the figure. ((a) and (a1)) White light images; ((b) and (b1)) fluorescence images.

At 1 h after tail-vein injection of tumor-bearing mice with FITC-iCREKA or the control fluorescent probe FITC-CREKA, tumor lesions and various organs and tissues were isolated and subjected to fluorescence imaging. Significant uptake of FITC-iCREKA was observed in tumors, whereas only a low level of fluorescence uptake was detected in normal brain tissues, yielding a tumor/brain ratio of 3.27 ± 0.78. The fluorescent probe FITC-iCREKA was eliminated via biliary, intestinal, and urinary excretion. Consistently, fluorescence largely accumulated in the gallbladder, intestinal tract, and both kidneys. Only low levels of fluorescence were distributed in other organs and tissues. Compared with the control fluorescent probe FITC-CREKA, tumor lesions took up considerably higher amounts of FITC-iCREKA. In addition, significant differences were observed in the tumor/brain ratio between FITC-iCREKA and FITC-CREKA (3.27 ± 0.78 versus 1.63 ± 0.68, *t* = 5.067, *P* ⩽ 0.001) (Figure 9).

### 3.7. The Tumor-Targeting Activity of ^18^F-iCREKA in Tumor-Bearing Mice

**Figure 10:**
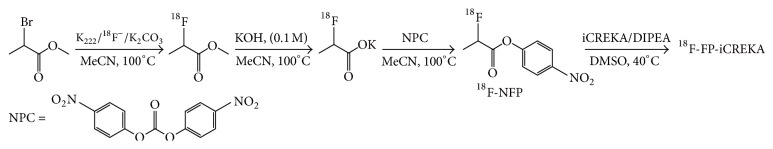
Schematic synthetic procedure of ^18^F-FP-iCREKA.

**Figure 11:**
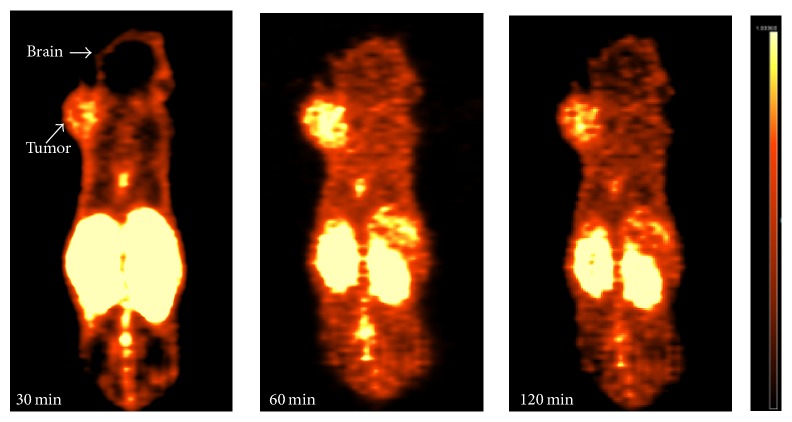
MicroPET/CT images at 30, 60, and 120 min after tail-vein injection of nude mice bearing U87 gliomas with ^18^F-iCREKA.

^18^F-iCREKA was successfully prepared by acylation. 4-Nitrophenyl-2-18F-fluoropropionate (^18^F-NFP) is a reaction precursor. Its nitrophenyl group can react with amino group of peptide, which can then form an amide bond between 2-^18^F-fluoropropionate and peptide. As a result, ^18^F was coupled to the peptides. The synthetic procedure was shown in Figure 10. The overall reaction time was 180 min. After HPLC purification, the radiochemical purity of ^18^F-iCREKA reached 97.0%. From the PET images in Figure 11, the tumor could be clearly visualized because the radioactivity uptake was significantly higher than that of the neighboring normal tissues. During the imaging process, although the uptake in tumor decreased slightly over time, the visualization of tumor was not obviously affected. High radioactivity distribution was also noted in both kidneys, which indicated 18F- iCREKA was eliminated mainly via the urinary system. The radioactivity distribution in the liver, gallbladder, and intestine was not very high, which implied that the hepatobiliary system was not the main elimination route of ^18^F- iCREKA. The radioactivity in the brain, head and neck, lungs, heart, and muscle was also minimal. Low radioactivity uptake in the brain contributed to a high tumor/brain ratio of 2.11 ± 0.24 at 30 min, 3.53 ± 0.31 at 60 min, and 3.04 ± 0.37 at 120 min, respectively. However, low tumor/kidney (0.88 ± 0.11 at 60 min) was observed.

## 4. Discussion

In the present study, the cyclic peptide iCREKA was successfully synthesized and prepared as a fluorescent and PET probe. Cytological examination, histological examination, and tumor imaging demonstrated that iCREKA-based molecular probes specifically targeted the fibrin-fibronectin complexes abundantly present in the tumor stroma. In addition, these molecular probes were able to achieve a tumor cell-penetrating effect after cleavage by MMP-2. Fluorescence and PET imaging showed that iCREKA-based molecular probes have the potential to be developed into molecular probes for tumor imaging.

The development of molecular probes capable of specifically targeting tumors holds great significance for tumor diagnosis, tumor staging, and the evaluation of therapeutic efficacy. Currently, ^18^F-fluorodeoxyglucose (^18^F-FDG) is the most commonly used molecular probe in the diagnosis and staging of malignant tumors and the evaluation of therapeutic efficacy. The application of ^18^F-FDGPET/CT imaging has a significant impact on the diagnosis and treatment of tumors [23–27]. However, ^18^F-FDG is a glucose analog and is therefore not a tumor-specific imaging agent. Some benign lesions such as inflammation, tuberculosis, and granulomatous lesions show increased metabolism, which may cause false positive results in ^18^F-FDGPET/CT imaging. Therefore, certain limitations exist in the application of ^18^F-FDG to diagnose tumors. A number of other imaging agents have been gradually developed. However, these imaging agents have been unable to surpass ^18^F-FDG and achieve wide recognition in clinical practice [28–30].

Studies have shown that fibrin-fibronectin complexes are abundantly present in the tumor stroma. The levels of fibrin-fibronectin complexes are significantly higher in the tumor stroma than in normal tissues. The above findings lay a foundation for studies that focus on targeting the tumor stroma. Fibrin-fibronectin complexes are expected to become targets for tumor stroma imaging. Work by Simberg et al. has shown that the small polypeptide CREKA is capable of specifically targeting fibrin-fibronectin complexes and thus possesses tumor-homing functions [8, 31, 32]. However, it is theoretically impossible to achieve tumor-specific targeting by simply targeting the fibrin-fibronectin complexes in the tumor stroma. In cases of inflammatory healing and trauma, large amounts of fibrin-fibronectin complexes often accumulate in the lesioned tissues. Thus, distinguishing tumors from inflammation is an important obstacle commonly facing tumor-imaging agents. In addition, as a linear polypeptide, CREKA is readily degraded by soluble peptidases present in the blood. The* in vivo* stability of CREKA is therefore much lower than that of the cyclic peptides.

Dual-target recognition is expected to achieve better tumor-specific targeting and be more conducive to distinguishing tumors from inflammation. Numerous studies have shown that MMP-2/9 are highly expressed in tumor tissues, which promotes the formation, repair, and maturation of tumor neovasculature and stimulates tumor growth [33–37]. MMP-2/9 is expressed at lower levels in normal tissues. Targeting MMP-2/9 in tumor tissues has been attempted in targeted cancer therapies [36–38]. In the present study, the polypeptide iCREKA was designed to incorporate an MMP2/9-cleavable linker. Consequently, specific penetration of iCREKA into tumor cells can be achieved only after iCREKA is cleaved by MMP-2/9. Therefore, iCREKA possesses dual-targeting properties, which is conducive to the enhancement of targeting specificity. In the presence of activated MMP-2, the accumulation of green fluorescence within the tumor cells was intense; however, in the absence of activated MMP-2, the uptake of green fluorescence within the tumor cells was minimal, which indicated that Tat mediated cellular internalization occurred in the presence of activated MMP-2, but not in the absence of activated MMP-2. The very slightly green fluorescence within the tumor cells in the absence of activated MMP-2 might be due to the self-luminescence of the substance within the cells, but not the cellular internalization. Dual targeting of fibrin-fibronectin complexes and MMP-2/9 in tumor tissues lays the foundation for achieving tumor-targeting and tumor-specific cell penetration.

The development of molecular probes that are taken up only by tumor cells and not by normal cells or cells with benign pathological changes is the basis for achieving tumor-specific imaging and targeted cancer therapy. Tumor-specific cell penetration is particularly important for targeted cancer therapies, especially for therapies designed to damage specific organelles in tumor cells. The tumor cell membrane often represents an insurmountable physiological barrier to a large number of drugs, and only after crossing this physiological barrier may tumor-cell-organelle-targeting drugs exert their effects. Therefore, tumor cell penetration has been a hot topic in cancer research. The present study showed that the addition of MMP-2 to the medium during incubation of tumor cells with FITC-iCREKA allowed FITC-iCREKA to penetrate into tumor cells. FITC-iCREKA not only accumulated in the cytoplasm of tumor cells but also reached the nucleus. At 3 h after intravenous injection of FITC-iCREKA into tumor-bearing mice, a large amount of fluorescence accumulated in the tumor cells but not in the tumor stroma. The above results indicated that the combination of dual targeting of fibrin-fibronectin complexes and MMP-2/9 and MMP-2/9-mediated cleavage allowed iCREKA to achieve tumor cell-specific targeting and membrane penetration. Fluorescence and PET imaging of tumor-bearing mice revealed significant uptake of the imaging probe by tumors and the accumulation of the imaging probe in normal pathways of excretion. In contrast, no apparent accumulation of the imaging probe was detected in other normal tissues in tumor-bearing mice. The results of the present study indicate that iCREKA has the potential to serve as a tumor-specific imaging agent as well as a tumor-targeting cell-penetrating carrier. It is expected that iCREKA can overcome the cell membrane barrier and enter cells freely while carrying therapeutic drugs, thereby playing a role in targeted cancer therapy.

Although the present study has demonstrated to a certain extent that iCREKA has the potential to serve as a tumor-targeting imaging agent or a carrier for targeted drugs, the following aspects of the PET imaging study need to be further addressed in detail: (1) iCREKA-based PET imaging should be conducted on a large number of tumor models to demonstrate repeatability. (2) iCREKA-based PET imaging should be conducted on other types of tumors besides glioma to determine the feasibility of broad-spectrum application of iCREKA. (3) In this study, ^18^F-iCREKA was synthesized by acylation. The labeling process was time consuming, and the yields were fairly low. The development of other labels to enhance synthetic yields and reduce the reaction time is a difficult issue that needs to be solved in future studies. Previous reports used a backbone-modified version of CREKA in order to increase the activity* in vivo*, such as David [39] that transformed CREKA into pocket-like shape to increase its accumulation in tumors. However, in the present study, we have not studied the differences on tumor PET imaging between these two different versions. Although we have confirmed the feasibility of tumor imaging of 18F-iCREKA on PET in the present study, further research is warranted to determine the superiority of the two versions of CREKA.

## 5. Conclusions

*In vivo* and* in vitro* experiments showed that iCREKA was able to target fibrin in the tumor stroma and subsequently penetrate into glioma cells, allowing successful tumor imaging. MicroPET/CT imaging demonstrated that ^18^F-iCREKA specifically targeted glioma lesions, which enables the visualization of the tumors.
